# Exploring the impact of surgical treatment for lung cancer in patients with Airway Obstruction from a Lung Cancer Screening Program

**DOI:** 10.1371/journal.pone.0320704

**Published:** 2025-05-08

**Authors:** Juan P. de-Torres, Juan José Girón-Flamenco, María Rodríguez, Alejandra de la Fuente-Añó, Valerio Perna, Miguel Mesa-Guzmán, Diego Murillo, Ana Belén Alcaide, Arancha Campo, Javier J. Zulueta, Gorka Bastarrika, Ana Ezponda, María del Mar Ocón, Carmen Felgueroso, Jesús Pueyo, Dolores Lozano, Luis M. Montuenga, Juan Berto, Teresa Perez-Warnisher, I. Madeleine Di-Frisco, Luis M. Seijo

**Affiliations:** 1 Pulmonary Department, Clínica Universidad de Navarra, Pamplona, Spain; 2 Cancer Center Clinica Universidad de Navarra (CCUN), Pamplona, Spain; 3 Instituto de Investigación Sanitaria de Navarra (IdiSNA), Pamplona, Spain; 4 Thoracic Surgery Department, Clínica Universidad de Navarra, Madrid, Spain; 5 Thoracic Surgery Department, Clínica Universidad de Navarra, Pamplona, Spain; 6 Division of Pulmonary, Critical Care and Sleep Medicine/Department of Medicine, Mount Sinai Morningside Hospital, Icahn School of Medicine at Mount Sinai, New York, New York, United States of America; 7 Radiology Department, Clínica Universidad de Navarra, Pamplona, Spain; 8 Pathology Department, Clínica Universidad de Navarra, Pamplona, Spain; 9 Solid Tumors Program, Center of Applied Medical Research (CIMA), Cancer Center Clinica Universidad de Navarra (CCUN), University of Navarra and IDISNA, Pamplona, Spain; 10 CIBERONC, Madrid, Spain; 11 Pulmonary Department, Clínica Universidad de Navarra, Madrid, Spain; University of Torino, ITALY

## Abstract

**Introduction:**

Little information is available on the surgical treatment options for patients with Airway Obstruction (AO) and early-stage non-small cell lung cancer (NSCLC) followed in lung cancer screening programs (LCS). This study aims to compare the potential impact of anatomical sub lobar resections vs. lobectomies in these patients.

**Methods:**

This is a retrospective analysis of participants who underwent surgical resections within a Lung Cancer Screening Program, including those with AO (post bronchodilator FEV_1_/FVC < 0.70). The short-term survival, locoregional recurrence, perioperative complications, and difference between pre and postoperative pulmonary function tests were compared between the surgical groups in those with AO.

**Results:**

Anatomical sub lobar resections or lobectomies for Stages IA and IB NSCLC were performed in 133 patients. Out of these, 57 had AO. Anatomical sub lobar resections were non-inferior to lobectomies for short-term survival in patients with AO (3-year survival rate: 95.8% vs. 97%, p = 0.83). In these patients, sub lobar resections had a higher recurrence rate (12.5% vs 0%, p < 0.01). No significant differences were found in postoperative complications between surgical techniques (sub lobar 33% vs lobectomy 24%, p = 0.44). Lastly, no significant difference was found on the change between pre and postoperative FEV_1_ and DLCO (p = 0.96 and 0.79 for FEV_1_ and DLCO, respectively).

**Conclusions:**

The present retrospective analysis suggests that sub lobar resection might be the best surgical option for treating early-stage NSCLC in patients with AO, where lung function preservation techniques are desired, but requires closer follow up to detect recurrence. Further studies in larger samples should confirm our findings.

## Introduction

A recent update on the Global initiative for Obstructive Lung Disease (GOLD) recommended that patients with Chronic Obstructive Pulmonary Disease (COPD) – defined as active or former smokers with airway obstruction (AO): FEV_1_/FVC ratio <0.7- should be included in lung cancer screening programs (LCSP) [1]. Those patients with COPD included in LCSP usually develop early-stage non-small cell lung cancer (NSCLC) that needs surgical treatment with excellent long-term results [2]. Due to their limited lung function and the possibility of developing a second lung cancer (LC), lung function sparing techniques are desired when treating these high-risk patients [2].

Lobectomies are the gold standard based on historical studies [3,4], but contemporary developments, including the Japan Clinical Oncology Group (JCOG) [5] and Cancer and Leukemia Group B (CALGB) trials [6], challenge this standard. Both studies concluded that sub lobar resections are, in fact, not inferior to lobectomies when dealing with small-sized (≤2 cm with a consolidation-to-tumor ratio >0.5) peripheral NSCLC regarding overall survival [5,6].

To our knowledge, there is no information in the literature that explores the potential impact of these surgical techniques for the curative treatment of patients with AO included in LCSP.

This study aims to compare anatomical sub lobar resections with lobectomies in terms of short-term survival, locoregional recurrence, perioperative morbidities, and difference between pre and postoperative pulmonary function tests in patients with AO participating in a LCSP. To explore if the results are different, we also included the comparison in those without AO.

## Methods

This was a retrospective analysis of a prospectively recruited group of patients that underwent surgery within the Pamplona International Early Lung Cancer Action Program (P-IELCAP) [7] between 2018–2022 and followed annually. Its population consisted of patients treated with either an anatomical segmentectomy or a lobectomy for stages IA and IB NSCLC.

The primary endpoint of the study focused on comparing short-term survival between anatomical sub lobar resections and lobectomies in those with AO.

AO was defined according to the GOLD guidelines [1] by the presence of a post-bronchodilator forced expiratory volume in the first second (FEV1)/forced vital capacity (FVC) <0.7 after 400 μg of inhaled salbutamol and classified according to their degree of severity: GOLD 1 FEV_1_% ≥ 80%, GOLD 2 FEV_1_% 50–79%, GOLD 3 FEV_1_% 30–49 and GOLD 4 FEV_1_% ≤ 29%.

Short-term survival was examined across two different timeframes: immediate post-operative mortality (encompassing any death related to the surgical intervention within the initial 90-day period following the procedure) and 3-year survival rates.

Secondary endpoints of the study include rates of locoregional recurrence, perioperative morbidities, and predicted postoperative pulmonary function tests (PFTs). Locoregional recurrence was defined as any relapse of the primary tumor within the ipsilateral thorax during the 3-year follow-up period. This recurrence includes the adjacent parenchyma, bronchus, ipsilateral hilar and/or mediastinal lymph nodes, thoracic wall, pleura, or malignant pleural effusion. Patients with positive margins in the definitive pathology report were excluded from the study. Perioperative complications were defined as any complication arising during the intervention or within the immediate 30-day postoperative period. Postoperative pulmonary function tests were determined by assessing FEV_1_ and diffusion capacity for carbon monoxide (DLCO) values for the patients following the surgical intervention at the 1-year follow-up appointment.

Additional endpoints include surgical approach, tumor diameter, consolidation-to-tumor ratio (CTR), perioperative complications, days with chest tube (CT), and discharge day. The surgical approach comprised three techniques: open (classical) thoracotomy, VATS, or RATS. The tumor diameter was defined as the largest diameter of the pulmonary nodule obtained from the pre-operative CT scan. The CTR was defined as the ratio between the nodule’s solid portion and subsolid portion, categorized as either >0.5 or < 0.5, also measured in the pre-operative CT scan. Any unexpected adverse events within the surgical procedure and 5–7 days after the intervention were considered perioperative complications. Prolonged air-leak was defined as a constant air leak lasting more than 5 days after the intervention. Days with chest tube were calculated from the day of the intervention to the day it was pulled, while the discharge day was calculated from the day of the intervention to the day the patient was discharged from the hospital. Although these factors were not the primary determinants in establishing the non-inferiority of anatomical sub lobar resections compared to lobectomies in treating early-stage NSCLC, their clinical significance warranted their inclusion in the study.

Anatomical sub lobar resections or lobectomies performed for known metastatic disease, small cell carcinomas, inflammatory conditions, or any other benign conditions were inherently excluded from this study. Likewise, eligible patients had to have no history of previous ipsilateral surgical interventions, chemotherapy, or radiotherapy for any other malignant diseases.

### Surgical techniques

Preoperative diagnosis was attempted in all cases. Surgical technique was decided based on each patient’s baseline lung function (FEV_1_ and DLCO% of predicted) and functional status.

Anatomical segmentectomies were defined as any sub lobar resection that included the sectioning of the segment’s corresponding vein, artery, and bronchus. These include single segmentectomies, bisegmentectomies (adjacent segments), culmenectomies (left S1 + S2 + S3), and basilar segmentectomies (S7-S10). Wedge or non-anatomical resections were excluded from the study, as were segmentectomies coupled with lobectomies, whether performed within the same operative procedure or in a prior or subsequent intervention.

Lobectomies were defined as any surgical intervention resulting in the removal of an entire pulmonary lobe, excluding those coupled with segmentectomies performed within the same procedure or in a prior or subsequent intervention. Bilobectomies, sleeve lobectomies, and pneumonectomies were excluded from this study.

In both groups, a systematic lymph node dissection was mandatory. This dissection had to include a sampling of at least three lymph node groups (e.g., groups 10, 7, and 4) for both groups. Lymph node involvement identified in the review of the pathological report implied exclusion from the study, since this implies that it is no longer an early-stage NSCLC.

The approach through which the surgical intervention was performed was not considered for inclusion or exclusion in the study. Either classical (open thoracotomy), Video-Assisted Thoracoscopy (VATS), or Robotic-Assisted Thoracoscopy (RATS) were valid approaches and deemed ontologically correct for the treatment of early-stage NSCLC [8–10]. Likewise, intraoperative conversion from a minimally invasive approach to a classical one was not considered sufficient for exclusion as long as all other inclusion criteria were met.

### Data collection

The University of Navarra’s ethics committee approved the study protocol and subsequent revisions (CUN CEIC 04/05/2000; current ref: 028/2012 mod3, 02/06/2022), and all subjects signed an informed consent prior to enrollment. For the present study data was accessed on March 1^st^ 2023.

We reviewed all patients who underwent surgical procedures from January 1^st^, 2018, to December 31^st^, 2022 within the P-IELCAP cohort. The initial data collection was followed by an analysis based on the surgical technique used. Specifically, only patients who underwent either a lobectomy or an anatomical sub lobar resection, as defined previously, were included. The surgical note was revised to confirm that the correct procedure with an adequate lymphadenectomy was performed.

Subsequently, a secondary analysis was conducted based on the initial inclusion criteria for the study: patients with suspected primary early-stage NSCLC on pre-operative CT scans and no evidence of lymphatic or distant metastases on PET scans. Exclusion criteria involved patients diagnosed with small cell carcinoma, metastasis, or any other benign condition before the intervention, ensuring that surgical interventions were conducted under suspicion of primary NSCLC.

To mitigate the possibility of false positives in the CT and PET scans, particularly for smaller tumors, a thorough review of the post-operative pathological reports was made as a secondary filter for inclusion. This process excluded patients diagnosed with conditions other than primary NSCLC in their definitive pathological report, as well as those with a more advanced staging.

Once the patients with primary NSCLC were confirmed, their AO status was investigated through their preoperative PFTs. Any patient with a post bronchodilator FEV_1_/FVC ratio <0.70 was considered to have AO and therefore grouped together within the corresponding sub lobar or lobar resection group.

Upon finalizing the selection of eligible patients, the different study endpoints were gathered through the patients’ digital history. To this end, a review of their medical and surgical history was necessary, along with an analysis of preoperative imaging tests and pulmonary function tests. The data used for the study was obtained through the digital history system of Clinica Universidad de Navarra.

### Statistical analysis

To explore the normality of the data distribution of the evaluated parameters, we used the Kolmogorov-Smirnov test. Data was summarized as relative frequencies for categorical variables and median (25^th^-75^th^ percentiles) for non-normally distributed variables. Chi-squared tests were performed to assess statistical significance for comparison of survival rates, locoregional recurrence and perioperative morbidities in patients with AO. For informative interest these comparisons were also done in patients without AO. An independent t-student test was performed for the change in PFTs between groups to determine statistical significance. Significance level was established as a two-tailed p-Value ≤0.05. We used SPSS 26.0, Chicago, U.S.A. for the statistical analysis.

## Results

**Fig 1:**
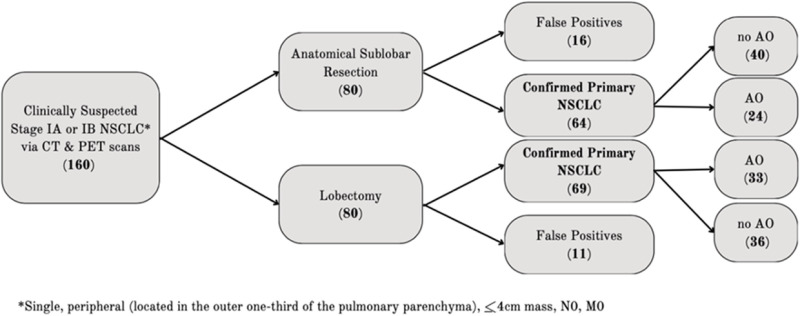
Inclusion algorithm. NSCLC: Non-small-cell Lung Cancer; AO: airway obstruction.

Between January 1^st^, 2018, and December 31^st^, 2022, 160 patients were initially gathered, 80 had undergone a lobectomy and 80 an anatomical sub lobar resection, all under suspicion of an early-stage primary NSCLC and followed for a mean of 2.5 years. **Fig 1** shows the algorithm for inclusion in the study. A total of 27 patients were excluded on a secondary analysis due to false positivity on the postoperative pathological report—11 (14%) from the lobectomy group and16 (20%) from the anatomical sub lobar resection group. These false positives were mainly inflammatory conditions, undiagnosed metastatic disease, hamartomas, or other benign pathologies.

Within the sub lobar group, 24 (38%) patients were confirmed to have AO, and 33 (48%) patients in the lobectomy group.

Of the 133 included patients, 84 (63.2%) were men, the average age was 67 (range 32–83), and 101 (81.2%) had a positive smoking history with a 56 pack-year average (range 5–164). On pre-operative imaging, the mean tumor diameter was 1.85 cm (range 0.4–4.0 cm) with 78 (58.6%) having a consolidation-to-tumor ratio greater than 0.5. With respect to the surgical approach, 16 (12.0%) underwent an open thoracotomy, 80 (60.2%) VATS, and 37 (27.8%) RATS.

**Fig 2:**
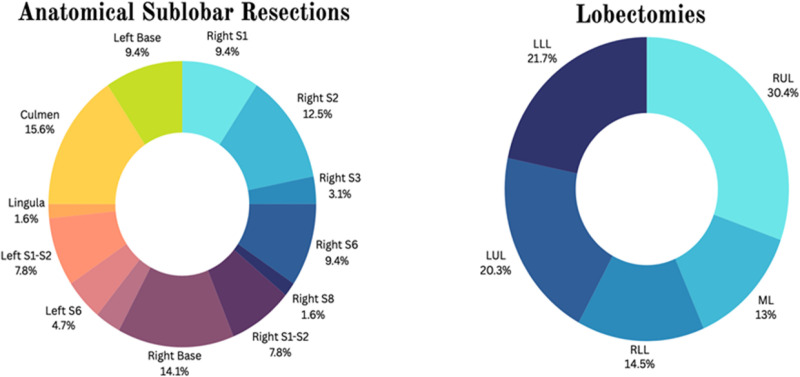
Surgical procedure performed in each group. S: segment; RUL: Right Upper Lobectomy; ML: Middle Lobectomy; RLL: Right Lower Lobectomy; LUL: Left Upper Lobectomy; LLL: Left Lower Lobectomy.

**Fig 2** depicts the distinct surgeries performed in each group.

Following the review of the definitive pathological report, 95 (71.4%) patients had an adenocarcinoma, 31 (23.3%) had squamous cell carcinoma, and 7 (5.3%) a typical carcinoid tumor. Of these, 63 (47.3%) were nodules between 1 and 2 cm and therefore classified as pT1b (Stage IA). Only 9 (6.8%) patients were Stage IB (T2a — tumor diameter between 3 and 4 cm).

**Table 1** illustrates the clinical and physiological characteristics of the participants stratified by AO status and surgical technique used.

**Table 1:** Clinical and physiological characteristics of the participants stratified by AO status and surgical technique used.

	Lobectomy + Airway Obstruction n = 33	Sub Lobar Resection + Airway Obstruction n = 24	Lobectomy + No Airway Obstruction n = 36	Sub Lobar Resection + No Airway Obstruction n = 40
**Age**, years	72 [54-83]	70 [51-79]	62 [34-80]	67 [32-83]
**Sex**				
Male	27 (82%)	19 (79%)	20 (56%)	18 (45%)
Female	6 (18%)	5 (21%)	16 (44%)	22 (55%)
**Smoking History**				
Yes	33 (100%)	21 (88%)	24 (67%)	30 (75%)
Pack-Years	43 [15-100]	48 [15-164]	39 [5-85]	27 [7-60]
No	0 (0%)	3 (12%)	12 (33%)	10 (25%)
**CTR**				
<0.5	13 (39%)	7 (29%)	11 (31%)	23 (58%)
>0.5	20 (61%)	17 (71%)	25 (69%)	17 (43%)
**Tumor diameter** (cm)	2.0 [0.7-4.0]	1.8 [0.9-3.6]	3.6 [0.6-3.9]	1.5 [0.4-2.5]
**Surgical Approach**				
Open Thoracotomy	7 (21%)	1 (4%)	7 (19%)	1 (3%)
VATS	23 (69%)	15 (63%)	26 (72%)	16 (40%)
RATS	3 (10%)	8 (33%)	3 (9%)	23 (57%)
**Pathological Diagnoses**				
Adenocarcinoma	21 (64%)	18 (75%)	24 (67%)	32 (80%)
Squamous cell carcinoma	12 (36%)	6 (25%)	6 (17%)	6 (15%)
Typical carcinoid	0 (0%)	0 (0%)	5 (14%)	2 (5%)
**TNM Staging**				
Tis/mi	4 (12%)	1 (4%)	0 (0%)	5 (13%)
T1a	5 (15%)	2 (8%)	9 (25%)	11 (28%)
T1b	12 (36%)	16 (67%)	5 (14%)	20 (50%)
T1c	8 (24%)	3 (13%)	19 (53%)	4 (10%)
T2a	4 (12%)	2 (8%)	3 (8%)	0 (0%)
**Days with CT**	4 [2–7]	2 [1–9]	3 [2–15]	2 [1–6]
**Discharge day**	4 [2–14]	2 [1–7]	4 [2–11]	2 [1–7]

CTR: consolidation-to-tumor ratio; VATS: Video-Assisted Thoracoscopy; RATS: Robotic Assisted Thoracoscopic; TNM: Tumor, Node, Metástasis; CT: chest Tube

Within patients with AO (43%), 46 (81%) were men, the average age was 71 (range 51–83), and 54 (95%) had a positive smoking history with a 46 pack-year average (range 15–164). On pre-operative imaging, the mean tumor diameter was 1.9 cm (range 0.7–3.6 cm) with 37 (65%) having a consolidation-to-tumor ratio greater than 0.5. The most common pathological diagnosis was adenocarcinoma (39, 68%) and most were pT1bN0M0 (28, 49%).

We also report the characteristics of those patients with no AO (57%). The majority were men (38, 51%) with an average age of 65 (range 32–83) and a smoking history (54, 71%) with an average of 33 pack-years (range 5–85). Within this group, the average tumor diameter was 2.3 cm (range 0.6–3.9) with 42 (55%) having a consolidation-to-tumor ratio >0.5. Similarly, adenocarcinoma was the most common pathological diagnosis (56, 74%), and most were pT1bN0M0 (25, 33%).

### Main outcomes

No significant (p = 0.59) differences were observed in the locoregional recurrence rates between both groups, despite anatomical sub lobar resections having more recurrences (5, 7.8%) than lobectomies (1, 1.4%, p < 0.05).

All patients (100%) survived the immediate post-operative period (90 days after the intervention), as well as 1-year after the procedure. Given the timeframe of the study, only 69 (51.8%) patients had a 3-year follow-up. From these, there was a similar 3-year survival rate in both groups—42 (95.4%) in the lobectomy group and 24 (96.0%) in the sub lobar group (p = 0.88).

Three deaths were reported, including 1 in the anatomical segmentectomy group and 2 in the lobectomy group. Of these, 2 (66.7%) were males older than 70 years with a positive smoking history, had airway obstruction, a CTR > 0.5, and a pathological diagnosis of a NSCLC other than an adenocarcinoma.

A total of 28 (21.1%) complications were reported. These immediate adverse effects were primarily due to prolonged air leaks in both groups—11 (15.9%) in the lobectomy group and 10 (15.6%) in the segmentectomy group.

Overall, the post-operative PFTs were 3% and 1% higher in the anatomical sub lobar resection group for FEV_1_ and DLCO, respectively. These changes were not statistically significant (p = 0.42 for FEV_1_ and 0.66 for DLCO).

Overall, AO patients had 16 (28%) complications and non-AO patients had 12 (16%) – p = 0.143.

**Table 2** shows the main outcomes of the study stratified by AO status and surgical technique.

**Table 2:** Outcomes of the study stratified by AO status and surgical technique.

	Lobectomy + Airway Obstruction n = 33	Sub Lobar Resection + Airway Obstruction n = 24		Lobectomy + No Airway Obstruction n = 36	Sub Lobar Resection + No Airway Obstruction n = 40	
**Short-Term Survival**						
90- days	33 (100%)	24 (100%)	–	36 (100%)	40 (100%)	–
1- year	33 (100%)	24 (100%)	–	36 (100%)	40 (100%)	–
3- years^*^	22/23 (96%)	11/12 (92%)	p = 0.64	20/21 (95%)	13/13 (100%)	p = < 0.01
**Locoregional Recurrence**	0 (0%)	3 (12.5%)	p < 0.01	1 (3%)	2 (5%)	p = 0.49
**PFTs** **(Postop – Preop)**						
FEV_1_	-3% [-21 - + 19]	-2.8% [-24 - + 7]	p = 0.96	-2.4% [-20 - + 24]	-1% [-12 - + 9]	p = 0.91
DLCO	-5.9% [-28 - + 6]	-7% [-22 - + 2]	p = 0.79	-5% [-25 - + 12]	-4.5% [-25 - + 18]	p = 0.72
**Perioperative complications**	8 (24%)	8 (33%)	p = 0.44	5 (14%)	7 (18%)	p = 0.14

*The *n* for the 3-year survival rate differs from the rest due to the timeframe of the study, which allowed for only a few patients to be followed up to 3 years.

PFTs: Pulmonary Function Tests; FEV_1_: Forced Expiratory Volume in the first second; DLCO: Diffusion capacity for carbon monoxide

### Patients with AO

Short term survival in the 3 different periods measured (90 days, 1 and 3 years) was no different by technique. Locoregional recurrence was higher in the sub lobar resection group. Mortality was similar by group techniques. One (8%) patient died in the sub lobar group and 1 (4.3%) in the lobectomy group (p = 0.64). In terms of lung function, there was no statistically significant difference between pre and postoperative PFTs (P = 0.96 and 0.79 for FEV_1_ and DLCO, respectively) for either surgical group. Within AO patients, those who underwent sub lobar resections had the same number of complications compared to their lobectomy counterparts (8 complications in each group, 33% and 24%, respectively; p = 0.44).

### Patients without AO

Short term survival was similar in both techniques although significantly higher at 3 years for sub lobar resections. No differences were found in locoregional recurrence with 5% of sub lobar resections, compared to 3% in the lobectomy group (p = 0.49). As for mortality, only 1 (4.7%) patient died in the lobectomy group (p < 0.01).

When evaluating post operative lung function changes, non-statistically significant changes were found between surgical techniques in this group (p = 0.91 and 0.72 for FEV_1_ and DLCO, respectively).

Lastly, 7 (18%) patients undergoing sub lobar resections and 5 (14%) lobectomies had postoperative complications, with non-statistically significant difference (p = 0.14).

## Discussion

The present retrospective analysis of a prospectively recruited cohort of participants of a LCSP reveals that in patients with AO, sub lobar resections are as effective as lobectomies in treating early-stage non-small cell lung cancer (NSCLC) concerning short-term survival, with a higher recurrence rate and similar postoperative lung function values. The present study supports an optimistic future for treating early lung cancer with limited lung resections in this high-risk population.

Patients with AO participating in LCSP have an increased risk of developing NSCLC, a risk that increases even more if they have concomitant visually detected emphysema [11]. This led the Global Initiative for Obstructive Lung Disease (GOLD) to recently recommend in their guidelines [1] the inclusion of COPD patients in LCSP if they fulfill the inclusion criteria (age 50–75, 20 pack year history and being active smoker or former smoker for < 15 years).

Recent data shows that if patients with COPD are well selected, their participation in LCSP allows the diagnosis of NSCLC in early stages with a survival outcome compatible with a curative state (2). It also showed that many of them, due to their increased LC risk, if followed for a long period (10 years), developed a second LC. Therefore, in the treatment of these patients in LCSP in whom lung function (mainly FEV_1_ and DLCO) is sometimes limited, the use of parenchyma-preserving surgical techniques is crucial.

To our knowledge, this is the first study exploring the impact of different surgical techniques on short-term survival, locoregional recurrence, perioperative morbidities, and predicted postoperative pulmonary function tests in patients with AO participating in a LCSP.

With the benefits of sub lobar resections on the survival and recurrence rates of early-stage NSCLC under debate, two major multicenter, randomized, controlled trials were conducted. The Japan Clinical Oncology Group (JCOG) (4) and the Cancer and Leukemia Group B (CALGB) [5] aimed to provide further insights on the possibility of using parenchyma-sparing techniques for treating smaller and less solid NSCLC. Despite inherent differences in the materials and methods between these studies, both established the non-inferiority of sub lobar resections to lobectomies for patients with cT1aN0M0 NSCLC (≤2 cm in diameter and within the peripheral 1/3 of the pulmonary parenchyma). Cornell University conducted a comparative analysis of both studies, ultimately affirming that the robust findings from both trials are consistent and provide compelling evidence supporting sub lobar resections as a potential new standard of care for early-stage NSCLC. This review also highlighted the importance of an intra-operative lymph node assessment for the exclusion of possible metastatic disease. Therefore, ensuring negative margins and implementing a thorough lymph node dissection is essential when performing segmentectomies [4,5,12]. Interestingly both studies also found that sub lobar resections had a higher recurrence rate, recommending that patients that undergo this technique should be followed up very closely to avoid this poor outcome.

In response to this ongoing challenge, the European Society of Thoracic Surgeons (ESTS) has recently outlined critical recommendations regarding technical standards for performing segmentectomies for the treatment of primary early-stage NSCLC [13]. This expert consensus highlights specific benchmarks that an anatomic segmentectomy must meet to ensure optimal patient outcomes. It emphasizes the vital aspects of intraoperative margin evaluation and the meticulous exclusion of lymph node involvement [14].

The present retrospective analysis focused on including patients who underwent anatomic sub lobar resections adhering to the established recommendations. This criterion ensured a comprehensive and accurate comparison between anatomic sub lobar resections and lobectomies for the treatment of early-stage NSCLC in patients with AO.

The results of the study demonstrate that anatomic sub lobar resections are, in fact, non-inferior to lobectomies in terms of short-term survival. This is the case for patients with a suspected primary NSCLC that is Stage IA or IB (a nodule ≤4 cm located in the peripheral 1/3 of the pulmonary parenchyma) with no evidence of lymph node or distant metastases. Not only were anatomic sub lobar resections non-inferior when compared to lobectomies in terms of short-term survival, but they proved to be superior in non-AO patients. However, those who had AO and underwent a sub lobar resection had a significantly higher recurrence rate than their lobectomy counter parts as previous studies reported [4,5] in patients not classified by AO status. This was not true for non-AO patients, where no statistically significant difference was found.

Regarding change in postoperative pulmonary function tests, once stratified by AO diagnosis, those patients previously diagnosed with AO who underwent anatomical sub lobar resections, had neither statistically nor clinically better PFT outcomes than those diagnosed with AO who underwent a lobectomy.

When we compared postoperative complications, there were also no statistically significant differences within the groups. Similar numbers were found despite stratifying for surgical technique, being prolonged air leaks the most common complication found.

Lastly but importantly, in terms of mortality, most deaths occurred among males aged over 70, with a history of smoking, a CTR > 0.5, AO, and a pathological diagnosis indicating a NSCLC other than adenocarcinoma. Despite the alarming mortality rates associated with lung cancer, this study reveals that patients with clinical stage I peripheral NSCLC who undergo surgery with curative intent can anticipate a 100% immediate and 1-year postoperative survival rate, with a 3-year survival rate of over 95%.

Supported by current studies, these findings place anatomic sub lobar resections as a potential new gold standard in treating early-stage NSCLC, even encompassing nodules classified as stage IB (3–4 cm) [15,16]. In comparison to segmentectomies, lobectomies seem inherently more invasive. While no evidence suggests a significant superiority in postoperative pulmonary function tests, the less aggressive nature of sub lobar resections allow broader treatment options for potentially new LC that these patients could develop in the future due to their increased risk [9].

Considering these aspects when determining the most suitable approach will significantly impact the long-term survival outcomes for individuals with early-stage lung cancer. While the non-inferiority of anatomic sub lobar resections predominates, the greater recurrence rates within AO patients call for a stricter follow-up protocol to ensure once again the early detection of possible recurrence or the development of new pulmonary neoplasms. The individualization of each case when it comes to its approach is crucial, ensuring not only a curative measure for the patient but also allowing ample room for potential future treatment of other pathologies.

### Limitations

This study exhibits some limitations, primarily stemming from its limited duration, a consequence of the recent adoption of sub lobar resections in our center. The study’s 3-year span restricts the population size and may affect the generalizability of the findings. Additionally, the short follow-up duration prevents a comprehensive long-term analysis, thus limiting the assessment of overall survival and recurrence-free rates. The fact that wedge or non-anatomical resections were excluded could be a limitation, acknowledging that these techniques are also used for surgical treatment of early-stage NSCLC lesions in LCSP (15). Another limitation is its single center nature, a multicenter study could provide a stronger and more generalizable evidence. To be clinically strict we call these patients with AO instead of COPD patients because unfortunately we do not have the complete clinical information to confirm that they have clinical symptoms of the disease (cough, sputum production, dyspnea, respiratory infections) to qualify as GOLD defined COPD [1]. Lastly, we did not produce a sample size calculation. These constraints call for the necessity of continued and prolonged research to establish stronger evidence regarding survival outcomes.

## Conclusions

The present retrospective study, further contributes to this discourse by delving into anatomical sub lobar resections vs. lobectomies for early-stage NSCLC in AO patients, extending its scope to T2a tumors (<4 cm). Results revealed non-inferiority in short-term survival for sub lobar resections compared to lobectomies, with similar post operative lung function values in AO patients. AO patients who underwent sub lobar resections had a higher rate of recurrence suggesting that they should have a stricter follow up. The anatomical sub lobar approach can therefore be deemed adequate as the first line of treatment when dealing with early-stage non-small cell lung cancer in patients with AO, were individualizing each case management is essential, especially in preserving their lung function with same curative results and ensuring adequate and strict follow-up.

**Study question**

Is the impact of a sub lobar anatomical resection the same in terms of short-term survival, locoregional recurrence, perioperative morbidities, and postoperative pulmonary function tests, compared with lobar resection for lung cancer in patients with airway obstruction participating in a lung cancer screening program?

**Central Message**

This retrospective analysis suggests that sub lobar anatomical resections of early-stage non-small cell lung cancer in patients with airway obstruction are non-inferior in terms of short-term survival, but require closer follow up to detect recurrence.

**Perspective**

The present study suggests that sub lobar anatomical resections could be the new standard of care for surgical treatment of early-stage non-small cell lung cancer in patients with airway obstruction.
